# Role of voltage-gated chloride channels in epilepsy: current insights and future directions

**DOI:** 10.3389/fphar.2025.1560392

**Published:** 2025-03-28

**Authors:** Ming-Ming Ni, Jie-Yu Sun, Zheng-Qian Li, Jin-Chun Qiu, Chun-Feng Wu

**Affiliations:** ^1^ Department of Pharmacy, Children’s Hospital of Nanjing Medical University, Nanjing, China; ^2^ Department of Pharmacy, The First Affiliated Hospital of Nanjing Medical University, Nanjing, China; ^3^ Department of Pharmacy, Qinghai Maternal and Child Health Hospital, Xining, China; ^4^ Department of Neurology, Children’s Hospital of Nanjing Medical University, Nanjing, China

**Keywords:** chloride channels, epilepsy, ion channels, neuronal excitability, genetic variants

## Abstract

Epilepsy is a prevalent chronic neurological disorder characterised by recurrent seizures caused by excessive neuronal discharge. Disruptions in chloride ion homeostasis significantly affect neuronal excitability, and play a crucial role in the pathophysiology of this disorder. This review highlights the emerging importance of chloride voltage-gated channels in epilepsy, which has been largely underappreciated compared to cation channels. Recent studies have suggested that genetic alterations in chloride channels, such as CLCN1, CLCN2, CLCN3, CLCN4, and CLCN6, contribute to neuronal excitability and seizure susceptibility, with variations in these channels acting more as susceptibility factors than direct causes. However, there is a significant gap in the research on other chloride channels, particularly ClC-Ka, ClC-Kb, ClC-5, and ClC-7, whose roles in epilepsy remain underexplored. Future research should focus on these channels to better understand their contribution to the pathophysiology of epilepsy. The incorporation of genetic tests for chloride channel variants in clinical practice could provide valuable insight into the aetiology of epilepsy, leading to improved diagnostic and therapeutic strategies for affected individuals.

## 1 Introduction

Epilepsy is one of the most prevalent chronic neurological disorders, affecting approximately 0.4%–1.0% of the global population (Chen et al., 2023), with an estimated 50 million individuals affected worldwide. It is characterised by recurrent paroxysmal disruptions of brain function resulting from excessive neuronal discharge, with various aetiologies within the central nervous system (Collaborators, 2019). An imbalance in the distribution of the main ions inside and outside neuronal cells, sodium (Na^+^), potassium (K^+^), calcium (Ca^2+^), and chloride (Cl^−^), constitutes the basis for the formation of neuronal resting and action potentials (Oyrer et al., 2018). Neuronal activity, which manifests as action potentials, relies on the coordinated flow of charged ions through ion channels to modulate neuronal excitability. Ion channel genes are among the most studied in epilepsy research, with approximately 25% of monogenic inherited epilepsies being associated with variants in ion channel genes (Gao et al., 2022).

Cl^−^ is the second most abundant ion in the human body after Na^+^, constituting approximately 70% of the total anionic content in the extracellular fluid (Berend et al., 2012). This high chloride concentration is essential for various physiological processes, including regulation of osmotic balance, maintenance of acid-base homeostasis, and modulation of neuronal excitability (Berend et al., 2012; Raut et al., 2024). In particular, Cl^−^ plays a critical role in the central nervous system, where it is essential to maintain the delicate balance of neuronal excitability (Fernandez-Abascal et al., 2021). Chloride voltage-gated chloride channels (ClCs), encoded by the evolutionarily conserved CLCN gene family, are critical for modulating chloride concentration gradients and membrane potentials (Jentsch, 2015). Structural or functional abnormalities in chloride channels can alter this delicate balance, leading to altered neuronal excitability (Fahlke, 2001). Such disruptions are of particular concern in epilepsy, in which an imbalance between excitatory and inhibitory signals in the brain can increase the susceptibility to seizures.

While extensive research exists on cation channels like sodium, potassium, and calcium in epilepsy, the role of ClCs is an emerging area of interest, with growing recognition of their importance in this disorder (Oyrer et al., 2018; Reid, 2024). Previous studies have focused on epilepsy involving these cation channels because of their direct roles in action potential generation and neuronal firing. This review highlights the evolving understanding of the contribution of chloride channels to epilepsy, highlighting their underappreciated but significant role in modulating neuronal excitability and susceptibility to seizures.

## 2 The ClC family of chloride channels

ClC proteins were first reported in electric fish, which express a large number of voltage-gated chloride channels in the cells of an electric organ originating from the skeletal muscle tissue (White and Miller, 1979). The molecular identification of these channels remained challenging until the 1990s because of the absence of specific inhibitors and difficulties in biochemical purification techniques. A breakthrough came with the cloning of a chloride channel from *Torpedo marmorata*, which was later identified as ClC-0 by Jentsch et al., in 1990 and marked the inception of the ClC family of anion transport proteins (Jentsch et al., 1990; Imbrici et al., 2015).

Recent studies have elucidated the prevalence of ClC-type chloride channels across a wide range of organisms, with nine distinct ClC sequences identified in mammalian and human cellular environments. The ClC family of chloride ion channels can be categorised into three functional groups according to their amino acid homology (Chen et al., 2023): ClC-1, ClC-2, ClC-Ka, and ClC-Kb (Collaborators, 2019); ClC-3, ClC-4, and ClC-5; and (Oyrer et al., 2018) ClC-6 and ClC-7. Among these, the ClC-1, ClC-2, ClC-Ka, and ClC-Kb channels facilitate the passive flow of chloride ions across cell membranes, while ClC-3 to ClC-7 function as chloride-proton exchangers, transporting both chloride ions and protons in a 2Cl^−^/H^+^ stoichiometry (Sahly et al., 2024; Graves et al., 2008; Ludwig et al., 2013). These channels are crucial for signal transduction, ion homeostasis, intracellular transport, and lysosomal protein degradation, making them key players in epilepsy pathophysiology.

ClCs exhibit features of molecular architecture and gating mechanisms that are unprecedented in other types of ion channels (Jentsch and Pusch, 2018; Jentsch et al., 2005a). ClC proteins form two-pore homodimers, with each subunit comprising 18 alpha helices, followed by a cytosolic carboxy-terminus that contains two conserved cystathionine-β-synthase (CBS) domains. A distinguishing feature of ClC proteins is the presence of an independent pore within each subunit, indicating that their gating mechanisms are autonomous and do not depend on neighbouring subunits. The gating of ClC channels involves a fast gate that operates separately in each pore of the dimer, and a common gate that coordinates the opening and closing of both subunits simultaneously. The fast gate is regulated by a critical glutamate residue, whereas the specific location of the common gate, which potentially involves the CBS domains at the dimer interface, remains less clearly defined. Moreover, the voltage dependence arises not from charged residues in the protein, but rather from the coupling of gating to the movement of chloride ions within the pore. These distinct gating mechanisms and structural features contribute to the unique functions and regulation of ClC ion channels (Jentsch, 2015; Jentsch and Pusch, 2018; Stolting et al., 2014). Currently, mutations in the CLCN1, CLCN2, CLCN3, CLCN4, and CLCN6 genes have been reported to be associated with various forms of epilepsy (Table 1).

**TABLE 1:** Genetic variants in chloride channels associated with epilepsy.

Gene	Nucleotide change	Protein change	Molecular consequence	Inheritance	Functional category	Country of origin	Study
CLCN1	c.2926C>T	p. Arg976Ter	Nonsense	*De novo*	NA	Caucasian	Chen et al. (2013)
	c.870C>G	p. Ile290Met	Missense	*De novo*	NA	Italian	Licchetta et al. (2014)
	c.501C>G	p. Phe167Leu	Missense	Inherited	NA	American	Peddareddygari et al. (2016)
CLCN2	c.704G>A	p. Arg235Gln	Missense	Inherited	Loss of function	Tunisian	Saint-Martin et al. (2009)
	c.1730G>A	p. Arg577Gln	Missense	Inherited	Loss of function	German	Saint-Martin et al. (2009)
	c.481G>A	p. Gly161Ser	Missense	*De novo*	NA	Chinese	Xie et al. (2019)
CLCN4	c.1630G>C or G>A	p. Gly544Arg	Missense	*De novo*	Loss of function	American	Veeramah et al., (2013), Palmer et al., (2018)
	c.43_55del	p. Asp15Serfs*18	Frameshift	Inherited	Loss of function	Belgian	Hu et al. (2016), Palmer et al. (2018)
	c.1606G>A	p. Val536Met	Missense	Inherited	Loss of function	Anglo-Australian	Hu et al. (2016), Palmer et al. (2018)
	c.661C>G	p. Leu221Val	Missense	Inherited	Loss of function	Anglo-Australian	Hu et al. (2016), Palmer et al. (2018)
	c.1876dup	p. Ile626Asnfs*135	Frameshift	Inherited	NA	Kurdish	Palmer et al. (2018)
	c.823G>A	p. Val275Met	Missense	*De novo*	Loss of function	Anglo-American	Palmer et al. (2018)
	c.2152C>T	p. Arg718Trp	Missense	*De novo*	Loss of function	Anglo-American	Palmer et al. (2018), Zhou et al. (2018), He et al. (2021a)
	c.1601C>T	p. Ser534Leu	Missense	*De novo*	Loss of function	Northern European	Palmer et al. (2018)
	Delete maternal	NA	Intragenic copy number deletion	Inherited	Loss of function	Hispanic	Palmer et al. (2018)
	c.1363G>A	p. Val455Ile	Missense	Inherited	Loss of function	Chinese	He et al. (2021a)
	c.1595C>A	p. Thr532Lys	Missense	*De novo*	Loss of function	Chinese	He et al. (2021a)
	c.1873C>T	p. Leu625Phe	Missense	Inherited	Loss of function	Chinese	He et al. (2021a)
	c.2167C>T	p. Arg723Trp	Missense	Inherited	Loss of function	Pakistani	Abdulkareem et al. (2023)
	c.2044G>A	p. Glu682Lys	Missense	Inherited	NA	Chinese	Lin et al., (2024)
	c.1597G>A	p. Val533Met	Missense	Inherited	NA	Turkey	Sager et al. (2023)
	c.1645 A>C	p. Ile549Leu	Missense	Inherited	Loss of function	French-Canadian	Sahly et al. (2024)
	c.265G>A	p. Asp89Asn	Missense	Inherited	Gain-of-function	Lebanese	Sahly et al. (2024)
	c.1024G>A	p. Gly342Arg	Missense	Inherited	Loss of function	South-Asian	Sahly et al. (2024)
CLCN6	c.956G>A	p. Arg319Gln	Missense	Inherited	NA	Japanese	Yamamoto et al. (2015)
	c.1159G>A	p. Val387Met	Missense	Inherited	NA	Japanese	Yamamoto et al. (2015)
	c.748G>A	p. Gly250Ser	Missense	Inherited	NA	Japanese	Yamamoto et al. (2015)
	c.533A>C	p. Glu178Ala	Missense	*De novo*	NA	Chinese	Wang et al. (2017a), Peng et al. (2018)
	c.599A>C	p. Glu200Ala	Missense	*De novo*	NA	Chinese	He et al. (2021a)

## 3 The emerging role of ClCs in epilepsy

### 3.1 CLCN1 gene and epilepsy

The CLCN1 gene is located on chromosome 7q35 and encodes the ClC-1 protein, a plasma membrane-bound chloride channel known for its high Cl^−^ conductance (Jentsch, 2008). ClC-1 is primarily expressed in the muscle tissue, where it contributes significantly to resting chloride conductance across the muscle membrane. Mutations in the CLCN1 gene can lead to partial or complete loss of ClC-1 channel function, a well-known cause of myotonia congenita, thus designating ClC-1 as the skeletal muscle chloride channel (Steinmeyer et al., 1991). Notably, the presence of polymorphic alleles in the CLCN1 gene among patients with idiopathic epilepsy indicates the potential involvement of ClC-1 chloride channels in neurological disorders (Chen et al., 2013). Chen et al. analysed a cohort of patients with idiopathic epilepsy and reported a three-fold increase in nonsynonymous single nucleotide polymorphisms (SNPs) in the CLCN1 gene compared to control individuals. Among these, a heterozygous nonsense mutation was identified. Arg976Ter in the CLCN1 gene in a patient with myotonic dystrophy and generalised epilepsy, resulting in a truncation of the distal C-terminus of the protein. In particular, the functional implications of p. Arg976Ter mutation, which deletes the last 12 amino acids of the ClC-1 protein, a shorter segment than the well-characterise myotonia-associated p. Arg894Ter mutation (Chen et al., 2013), were not functionally assessed. Although the precise pathophysiological mechanisms of ClC-1 channel mutations in epilepsy remain unclear, studies have shown that overexpression of ClC-1 in inhibitory Purkinje cells hyperpolarises their resting membrane potential and reduces excitability (Lorenzetto et al., 2009). This alteration in excitability can lead to disinhibition of cerebellar nuclei, which could influence epileptogenesis (Kros et al., 2015). This study also showed the presence of ClC-1 mRNA transcripts and ClC-1 protein bands in various regions of the human brain, including the hippocampus, cerebellar Purkinje cell layer, brainstem nuclei, frontal neocortex, and thalamic nuclei. This novel localisation of ClC-1 may provide new perspectives on the role of ClC channels in the central nervous system (Chen et al., 2013). Furthermore, ClC-1 has also been detected in subcortical structures, such as the basal ganglia and subthalamus, raising the possibility that dysfunction of brain ClC-1 may contribute to the dystonia phenotype in individuals with CLCN1 mutations, which have traditionally been considered exclusively muscular in origin.

Despite these findings, patients with myotonia congenita and related animal models do not typically show central neurological symptoms and very few cases of CLCN1 mutations associated with seizures have been reported. Licchetta et al. were the first to describe a case of epilepsy and limbic encephalitis in a family with ClC-1-linked myotonia caused by p. Ile290Met mutation. Epilepsy in this patient was most likely attributable to limbic encephalitis associated with antibodies against glutamate decarboxylase (Licchetta et al., 2014). Similarly, Peddareddygari et al. reported the case of a 57-year-old American male with clinical features indicative of myotonic dystrophy type II accompanied by focal seizures. Genetic analysis revealed a mutation in the CCHC-type zinc finger nucleic acid binding protein (CNBP) gene associated with myotonic dystrophy, along with a heterozygous CLCN1 mutation p. Phe167Leu (Peddareddygari et al., 2016).

Mutations in the CNBP or CLCN1 genes, individually or in combination, contribute to the pathophysiology of seizures. Overall, although several studies have suggested a possible association between CLCN1 mutations and epilepsy, particularly through the presence of ClC-1 in the brain tissue, the current understanding remains limited. The rarity of seizures in patients with myotonia congenita and the low incidence of CLCN1 mutations linked to epilepsy indicate that a direct role of ClC-1 in epilepsy is unlikely. Additional research is required to elucidate the functional implications of CLCN1 mutations and their potential involvement in neurological conditions, particularly dystonia and other movement disorders.

### 3.2 CLCN2 gene and epilepsy

The CLCN2 gene is located on chromosome 3q27 and encodes the CLC-2 protein, which is ubiquitously expressed in the central nervous system in comparison to ClC-1. In the brain, ClC-2 is found in pyramidal neurons of the hippocampus, interneurons, as well as in astrocytes in the end feet surrounding blood vessels and in oligodendrocytes (Elorza-Vidal et al., 2019). A direct role of ClC-2 in the regulation of neuronal excitability has been proposed, where ClC-2 facilitates the compensatory efflux of chloride, maintaining low intracellular chloride concentrations during chloride loading caused by repetitive activation of hyperpolarizing γ-aminobutyric acid (GABA) receptors. This mechanism provides a plausible biological basis for how the loss of CLCN2 function could result in increased excitability in certain neurons (Ge et al., 2011). In oligodendrocytes, dysregulated ClC-2 may cause demyelination and disrupted ion balance, impairing axonal conduction and increasing neuronal excitability (Hou et al., 2018). The combination of demyelination and ion imbalance can enhance neuronal excitability and synchronize firing, thus facilitating the development of seizures. In addition, aged ClC-2 knockout mice exhibit altered neurotransmission patterns and heightened neuronal excitation, which are linked to astrocyte activation and neuronal degeneration (Cortez et al., 2010). Furthermore, linkage analysis identified a locus for IGE at 3q26, the chromosomal region harbouring CLCN2 (Sander et al., 2000). Research has indicated that the expression of CLCN2 in epilepsy-associated brain tissue is approximately 50% lower than that of controls, indicating a significant loss of functional ClC-2 channels (Bertelli et al., 2007). CLCN2 knockout mice were expected to exhibit an epileptic phenotype, as ClC-2 is believed to be essential to maintain low cytoplasmic chloride levels in GABAergic neurones. However, CLCN2 knockout mice do not exhibit spontaneous epilepsy or reduced seizure thresholds (Blanz et al., 2007; Rinke et al., 2010). One potential explanation for this phenomenon is that the hyperexcitability of some neurones is balanced by the increased excitability of local inhibitory interneurons (Rinke et al., 2010). Alternatively, the upregulation or downregulation of other Cl^−^ channels, such as ClC-1, might compensate for the deletion of ClC-2 (Rahmati et al., 2018). These adaptive mechanisms could help maintain the balance between excitatory and inhibitory activity within neural circuits, effectively counteracting the expected increase in neuronal excitability resulting from the absence of ClC-2.

The role of CLCN2 mutations in epilepsy remains controversial, with conflicting data complicating the interpretation of their functions. Previous studies in humans have suggested a potential link between CLCN2 mutations and epilepsy, particularly idiopathic generalised epilepsy (IGE) and focal epilepsy. An original study by Haug et al. reported heterozygous nonsense and missense mutations associated with IGE in three families (Haug et al., 2003). However, subsequent research, including that by Niemeyer et al., raised concerns about the pathogenicity of these mutations, as they did not alter ClC-2 function *in vitro* (Niemeyer et al., 2004). The study was retracted in 2009, with findings suggesting that these mutations were not pathogenic and were present in asymptomatic relatives. Additionally, it has been proposed that these mutations may serve as susceptibility factors rather than direct causes of epilepsy (Kleefuss-Lie et al., 2009). Further research identified additional variants, such as the intronic variant IVS17-3C>T and two missense variants (p. Arg688Gly and p. Glu718Asp) in patients with IGE (D'Agostino et al., 2004). However, these coding sequence abnormalities were also found in the control patients, suggesting that they are not exclusive to patients with epilepsy. Additionally, several large-scale studies have failed to detect significant CLCN2 mutations in cohorts of patients with IGE or other epilepsy syndromes (Blanz et al., 2007; Everett et al., 2007; Stogmann et al., 2006), and the causal role of CLCN2 mutations has been questioned by several authors, further complicating our understanding of the role of the gene in epilepsy. One argument against the direct link between CLCN2 mutations and epilepsy is that these mutations do not follow a Mendelian inheritance pattern. Many of these sequence variations exhibit incomplete co-segregation, appearing not only in affected individuals but also in unaffected members of the same family. In particular, a recent study by Saint-Martin et al. identified two novel missense mutations, p. Arg235Gln and p. Arg577Gln, in the CLCN2 gene in individuals with IGE. Although these variants did not completely co-segregate with epileptic phenotypes, their absence in controls and their functional implications suggest that they may contribute to epilepsy susceptibility in conjunction with other unidentified genetic factors (Saint-Martin et al., 2009). Similarly, the recent identification of p. Gly161Ser variant by Xie et al. highlighted the potential impact of specific mutations on the selectivity filter motif of the ClC-2 channel, which could alter its function and implicate CLCN2 more directly in the pathophysiology of IGE (Xie et al., 2019). This complexity suggests that epilepsy is not only the result of a single disease-causing mutation but also arises from the co-existence of multiple sequence variations across distinct genes. A polygenic heterogeneity model posits that although CLCN2 mutations can influence neuronal excitability, these changes likely lead to epilepsy only in conjunction with mutations in other genes (Klassen et al., 2011). The role of CLCN2 mutations in epilepsy appears to be nuanced, with these mutations possibly acting as susceptibility factors rather than direct causes.

### 3.3 CLCN3 gene and epilepsy

The CLCN3 gene is located on chromosome 4q33 and encodes the CIC-3 protein, which is widely expressed in various tissues and organs, with particularly high levels in the brain, especially in the hippocampus, a region known for its significant involvement in memory and learning, as well as its status as one of the most epileptogenic structures (Duran et al., 2010; Verkman and Galietta, 2009). ClC-3 is primarily localized to the membranes of endosomal and lysosomal vesicles, where it functions as a 2Cl^-^/H^+^ exchanger, playing a crucial role in vesicular acidification. Mutations in ClC-3 disrupt this acidification process, leading to the accumulation of chloride within the vesicles. This disturbance impairs the function of the proton pump, weakening the acidic environment and consequently affecting critical processes such as lysosomal protein degradation, vesicle fusion, and neurotransmitter trafficking, including GABA receptors. The resulting impairment of GABAergic signalling contributes to an imbalance between excitation and inhibition, which may increase susceptibility to seizures (Jentsch et al., 2005a; Dickerson et al., 2002). Research has indicated that elevated expression of CLC-3 has been observed in the hippocampus of both animal models and patients with temporal lobe epilepsy (TLE), suggesting a potential link between increased CIC-3 channel expression and epileptic conditions (Shen et al., 2021). Shen et al. highlighted this association, noting that higher levels of hippocampal CLC-3 expression correlated positively with the average absolute power of high-frequency oscillations (HFOs), which are electrophysiological markers often associated with epileptic activity. A correlation was also observed between CLC-3 expression and the number of ictal HFOs recorded during seizures in patients with TLE (48). In animal studies, CLC-3 knockout mice demonstrated reduced susceptibility to seizures induced by pentylenetetrazole, a chemical that can provoke seizure activity, and showed prolonged sedation in response to benzodiazepines, which enhances GABAergic signalling (Dickerson et al., 2002). Further investigation of neonatal neurones from CLC-3 knockout mice revealed lack of outwards-rectifying chloride currents and decreased epileptiform activity after stimulation. This reduction may be attributed to the blockade of CLC-3 chloride channels, which stabilise intracellular chloride homeostasis and alter GABA excitatory activity (Wu et al., 2019). It can be hypothesised that the CLCN3 gene may serve as a susceptibility factor or pathogenic gene in epilepsy. However, to date, no pathogenic mutations have been reported in the CLCN3 gene in patients with epilepsy.

### 3.4 CLCN4 gene and epilepsy

The CLCN4 gene, located on chromosome Xp22.2, encodes the CIC-4 protein, which is predominantly expressed in brain tissues (He et al., 2021a), particularly in the pyramidal cells of the cortex (the dentate gyrus of the hippocampus) and the Purkinje cell layer (Jentsch and Pusch, 2018; Park et al., 2017). Although the precise function of this gene remains unclear, it is believed to act as an electrogenic 2Cl^-^/H^+^ exchanger, and may play a role in the development of neurological disorders. CLCN4 mutations impair 2Cl^-^/H^+^ transport, disrupting ion homeostasis and normal cellular processes. This affects protein function, disrupts vesicular trafficking, and hinders neuronal differentiation, potentially contributing to neuronal excitability and epilepsy (Jentsch and Pusch, 2018; Hu et al., 2016). Moreover, CLCN4 has been implicated in X-linked intellectual disabilities and epilepsy, with prominent focal seizures observed in some cases. In 2013, a *de novo* loss of function mutation, p. Gly544Arg, in the CLCN4 gene was first reported to cause early-onset epileptic encephalopathy accompanied by severe developmental delays (Veeramah et al., 2013). Subsequently, five additional CLCN4 variants were identified, among which p. Asp15Serfs*18, p. Val536met, and p. Leu221Val were associated with seizures (Hu et al., 2016). Furthermore, the genotypic findings of Palmer et al. are consistent with, and extend beyond, those of previous studies, identifying an additional novel CLCN4 frameshift variant, p. Ile626Asnfs135 and three novel missense variants (p. Val275Met, p. Arg718Trp, and p. Ser534Leu) along with a novel intragenic microdeletion involving exon 12 (Palmer et al., 2018).

In particular, the p. Arg718Trp variant associated with epilepsy has also been reported in two additional studies (He et al., 2021a; Zhou et al., 2018). With advances in large-scale parallel sequencing, an increasing number of variants associated with various phenotypic features and degrees of disease severity have been reported. He et al. subsequently reported three novel variants (p. Val455Ile, p. Thr532Lys, and p. Leu625Phe) (He et al., 2021a), followed by several other mutations, including p. Arg723Trp (Abdulkareem et al., 2023), p. Glu682Lys (Lin et al., 2024), p. Val533Met (Sager et al., 2023), p. Ile549Leu, and p. Asp89Asn (Sahly et al., 2024). The phenotypic spectrum of CLCN4-related epilepsy includes medication-resistant seizures, intellectual disabilities, behavioural disorders, and congenital anomalies. In particular, patients with multiple seizure types or those with missense or *de novo* variants typically exhibit more severe phenotypes, whereas individuals with a single seizure type, frameshift or intragenic deletions, or inherited variants often present with milder symptoms. Electrophysiological studies have revealed that most of these missense variants reduce or abolish ClC-4 currents, which is consistent with the loss of function that underlies epilepsy and cognitive defects associated with these genetic alterations (He et al., 2021a). This dysfunction likely disrupts the normal electrochemical gradients and excitability of the neurones, contributing to the development of seizures and other neurological symptoms.

### 3.5 CLCN6 gene and epilepsy

The CLCN6 gene is located on chromosome 1p36 and encodes the CLC-6 protein, which is predominantly expressed in the brain. This protein functions as a 2Cl^−^/H^+^ exchanger and is primarily localized in late endosomes, playing a critical role in maintaining ion homeostasis within these cellular compartments. While disruption of CLCN6 in mice results in mild lysosomal storage, particularly in the axon initial segments, no definitive link between CLCN6 mutations and human disease has been established to date (Polovitskaya et al., 2020). Recent massive parallel sequencing studies in patients with sporadic epilepsy of unknown aetiology have identified several SNPs in chloride channel genes, including CLCN6 variants, which were absent in control subjects (Chen et al., 2013). Yamamoto et al. have reported three nucleotide variants in the CLCN6 gene (p. Arg319Gln, p. V387M, and p. Gly250Ser), which were believed to be associated with epilepsy and febrile seizures (Yamamoto et al., 2015). However, subsequent *in vitro* analyses assessing the functional relevance of these SNPs did not indicate significant differences in the activity of cells expressing the wild-type and mutant variants (Yamamoto et al., 2015). Furthermore, both studies reported that the *de novo* heterozygous mutation p. Glu178Ala in the CLCN6 gene is associated with West Syndrome, a severe form of epilepsy that typically presents in infancy and is characterised by developmental regression and specific seizure patterns known as infantile spasms (Peng et al., 2018; Wang Y. et al., 2017). In particular, He et al. were the first to identify the CLCN6 variant p. Glu200Ala as a pathogenic mutation associated with West Syndrome. Missense mutations can contribute to this condition by preventing the fusion of autophagosomes with lysosomes, thereby disrupting critical cellular degradation processes (He et al., 2021b). Overall, these studies suggest a potential association between the CLCN6 gene and epilepsy; however, observations from ClCN6 knockout mice indicate that these animals do not exhibit epilepsy or severe neurological defects (Poet et al., 2006). However, compensation by other chloride channels cannot be ruled out as a possible explanation for the moderate phenotypes observed in these knockout models. This highlights the complexity of the genetic contributions to epilepsy and the need for more research to fully elucidate the role of CLCN6 in neurological disorders.

## 4 Conclusion and future perspectives

There is a notable lack of genetic studies identifying mutations in ClC-Ka, ClC-Kb, ClC-5, and ClC-7 in patients with epilepsy, highlighting the significant gap in our understanding of the specific roles of these chloride channels in the mechanisms of epilepsy. ClC-Ka and ClC-Kb are primarily responsible for the regulation of chloride transport across various tissues, particularly in the kidneys, where they are essential for maintaining cellular ion balance and fluid homeostasis (Coppola et al., 2023). However, their direct roles in brain function and epilepsy have not been well characterised. Similarly, ClC-5 and ClC-7 contribute to endosomal chloride transport and are associated with various renal and skeletal pathologies (Zifarelli, 2015). Therefore, in future studies using targeted genetic screening and functional analyses, researchers can establish potential associations between mutations in ClC-Ka, ClC-Kb, ClC-5, and ClC-7, and susceptibility to epilepsy. Such investigations could significantly improve our understanding of the genetic foundations of seizure disorders, and inform the development of targeted therapeutic strategies.

The Na-K-2Cl cotransporter isoform 1 (NKCC1) and the K-Cl cotransporter isoform 2 (KCC2) are two key cation-chloride cotransporters implicated in human epilepsy, responsible for regulating chloride homeostasis by accumulating and extruding Cl^−^ (Liu et al., 2019). Dysfunction of ClCs, such as ClC-2, may indirectly affect the activity of NKCC1 and KCC2 by disrupting this delicate chloride balance. Impaired chloride channels lead to chloride accumulation within the cell, disrupting the chloride gradient. This disturbance impairs KCC2’s ability to effectively export chloride and exacerbates chloride accumulation through NKCC1. As a result, this imbalance can reverse the polarity of GABAergic signalling, shifting it from inhibitory to excitatory. The ensuing increase in neuronal excitability may contribute to the onset of seizures, particularly in epilepsy with developmental origins, as the reversion of NKCC1 and KCC2 expression to an immature state may be one of the key mechanisms underlying epileptogenesis (Blaesse et al., 2009). In addition to their role in chloride homeostasis, ClC channels, particularly ClC-2 and ClC-3, are involved in cell volume regulation by facilitating chloride and water transport (Duran et al., 2010; Wang H. et al., 2017). Neurons are particularly sensitive to changes in cell volume, and abnormal swelling can significantly impair their function. Swollen neurons may experience compression of synaptic components, altered ion gradients, and disruption of membrane potential, all of which enhance neuronal excitability. This increase in excitability can cause abnormal neuronal firing, thus heightening the likelihood of seizure generation (Wilson and Mongin, 2018). The dysfunction of ClC channels disrupts chloride transport, leading to an osmotic imbalance that promotes cell swelling, shifts inhibitory signalling to excitatory, and enhances neuronal hyperexcitability, ultimately contributing to the initiation and propagation of seizures. The intricate relationship between chloride channels and epilepsy has become increasingly apparent. Various chloride channel genes, including CLCN1, CLCN2, CLCN3, CLCN4, and CLCN6, are associated with epilepsy and other neurological disorders, indicating that disruption in chloride homeostasis can significantly affect neuronal excitability and susceptibility to seizures. Despite the emerging evidence linking specific mutations in these genes with epilepsy, the functional implications of many of these mutations remain unclear. For example, while some CLCN6 variants have been identified in patients, *in vitro* studies have not demonstrated significant functional differences compared to wild-type channels. This raises critical questions about how these mutations can contribute to the epileptic phenotype, and emphasises the need for further investigation to clarify the precise roles of these channels in neuronal function.

Compensatory mechanisms involving chloride channels suggest a complex interplay that mitigates the effects of specific mutations. Future research should focus on understanding these compensatory pathways and their effects on the clinical manifestations of epilepsy. Furthermore, phenotypic variability observed in patients with chloride channel gene mutations emphasises the need for comprehensive genetic testing and personalised treatment approaches. As the genetic landscape of epilepsy evolves, it will be vital to include chloride channel genes in the genetic testing panels for patients with undiagnosed epilepsy. Identifying mutations in these genes can provide valuable insights into the aetiology of epilepsy, guide management strategies, and improve patient outcomes.

Compared to voltage-gated cation channels, targeting ClCs for anti-epileptic therapies presents several unique challenges. Voltage-gated cation channels, such as Na^+^ or Ca^2+^ channels, are well-established therapeutic targets with a relatively clearer understanding of their mechanisms of action. In contrast, ClCs are involved in various cellular processes beyond just chloride homeostasis, such as volume regulation and neurotransmitter trafficking, making their manipulation more complex. A major concern is side effects, as ClCs are ubiquitously expressed in tissues like the brain, kidneys, and heart. Altering their function could lead to unintended consequences such as renal dysfunction, fluid balance disturbances, or cardiovascular issues (Jentsch et al., 2005b; Piwon et al., 2000; Duan, 2011), emphasizing the need for highly selective approaches that target brain-expressed ClC isoforms. Drug delivery is another significant challenge, as ClC channels are often localized in specific intracellular compartments, such as endosomes and synaptic vesicles. Effectively targeting these compartments requires advanced drug delivery methods, such as nanoparticle-based or lipid-based carriers, which must be capable of crossing the blood-brain barrier while maintaining efficiency and safety. Additionally, ClCs have multiple isoforms with overlapping roles in both neuronal and non-neuronal cells, making precise isoform-specific targeting more complex than with cation channels. Although significant progress has been made in understanding the role of chloride channels in epilepsy, ongoing research is essential to uncover the underlying mechanisms, explore potential therapeutic targets, and enhance the diagnosis and treatment of this complex disorder.

## Abbreviations

Cl^−^, Chloride ions; ClCs, Chloride voltage-gated channels; SNPs, Single nucleotide polymorphisms; GABA, Hyperpolarising γ-aminobutyric acid; HFOs, High-frequency oscillations; TLE, Temporal lobe epilepsy; IGE, Idiopathic generalised epilepsy; NKCC1, Na-K-2Cl cotransporter isoform 1; KCC2, K-Cl cotransporter isoform 2.
